# The neuroprotective effect of *Ocimum sanctum* Linn. ethanolic extract on human embryonic kidney-293 cells as *in vitro* model of neurodegenerative disease

**DOI:** 10.14202/vetworld.2018.1237-1243

**Published:** 2018-09-11

**Authors:** Puspa Hening, Made Bagus Mataram Auriva, Nastiti Wijayanti, Dwi Liliek Kusindarta, Hevi Wihadmadyatami

**Affiliations:** 1 Research Center of Biotechnology, Universitas Gadjah Mada, Yogyakarta, 55281, Indonesia; 2 Department of Anatomy, Faculty of Veterinary Medicine, Universitas Gadjah Mada, Yogyakarta, 55281, Indonesia; 3 Department of Animal Physiology, Faculty of Biology, Universitas Gadjah Mada, Yogyakarta, 55281, Indonesia

**Keywords:** choline acetyltransferase, human embryonic kidney-293, neurodegenerative diseases, *Ocimum sanctum* Linn. ethanolic extract

## Abstract

**Aim:**

This study aimed to analyze the neuroprotective effect of *Ocimum sanctum* Linn. ethanolic extract (OSE) on human embryonic kidney-293 (HEK-293) cells as the *in vitro* model of neurodegenerative diseases.

**Materials and Methods:**

In this research, HEK-293 cells divided into five groups consisting of normal and healthy cells (NT), cells treated with Camptothecin 500 µM as the negative control, cells treated with trimethyltin 10 µM (TMT), cells treated with OSE 75 µg/ml, and cells pre-treated with OSE 75 µg/ml then induced by TMT 10 µM (OSE+TMT). MTT assay and phase contrast microscopy were applied to observe the cell viability quantitatively and morphological after *Ocimum sanctum* Linn extract treatment. Finally, the reverse transcription polymerase chain reaction was employed to study the expression of choline acetyltransferase (ChAT).

**Results:**

The MTT assay and phase contrast microscopy showed that OSE pre-treatment significantly increased the viability of TMT-induced apoptotic cells and maintained cell viability of the normal HEK-293 cells. Expression of ChAT markedly reduced on TMT treatment group, but OSE administration stabilized ChAT expression in TMT-induced HEK-293 cells.

**Conclusion:**

This present study proved that OSE administration has neuroprotective effect by increased HEK-293 cells viability and maintain ChAT expression.

## Introduction

Neuronal cell death is the main feature of many human neurodegenerative diseases. It can affect the central nervous system causing the progressive decline of nervous system function [1,2]. The genetic factors and environmental exposure may trigger the progression of the diseases. At present, there are few curative treatments available for neurodegenerative conditions, even though some drugs have already licensed for many years, such as levodopa in Parkinson’s disease [3] and acetylcholinesterase inhibitors in Alzheimer’s disease, which improve the symptoms only in the early stage of these diseases [4]. Therefore, preventive and therapeutic strategies suggested for effective intervention [5].

Acetylcholine (ACh) is a neurotransmitter that plays a role in regulating the functional mechanism of neurons such as proliferation, differentiation, and apoptosis [6]. ACh is produced by cholinergic neuron, and it contributes to cognitive, learning, and memory mechanism [7]. Dysfunction of presynaptic cholinergic system marked by decreased choline acetyltransferase (ChAT) activity causes cognitive deficits [8,9]. Inhibition of ChAT production that contributes to the biosynthesis of ACh due to aging or pathology may interfere with ACh production. The decreased of ACh production will lead to the occurrence of neurodegenerative symptoms [10,11]. Therefore, ChAT can be a potential target or neurodegenerative diseases prevention and therapy.

*Ocimum sanctum* Linn. is a member of the family Lamiaceae (Labiatae). The members of family Lamiaceae have been proven as a traditional herb that shows a variety of biological activities. Biological studies on this plants have reported anti-inflammatory [12], antiallergic [13], antioxidant effect [14] and also the possibility for zootechnical and reproductive medicine applications [15]. Lamiaceae exhibits cytotoxic and apoptotic effects on human cervical carcinoma (HeLa) and human breast cancer (MCF-7) cell lines [16] and gastric carcinoma cells [17]. *O. sanctum* Linn. is herbal plants that distributed widely in Indonesia. It contains many nutrients and biologically active compounds. Liquid chromatography - electrospray ionization - mass spectrometry shows that fraction of *O. sanctum* Linn. composed of eugenol, luteolin, apigenin, and ursolic acid [18]. Those metabolites are potential to be explored for the drugs development.

Several studies using *O. sanctum* Linn. ethanolic extract (OSE) showed positive results on the ability of the central nervous system as the center of learning and memory [19]. Administration of OSE also promotes the density of pyramidal cells in the CA1 and CA3 mediated by increased concentration of ACh [20]. *O. sanctum* Linn. has been reported for its protective effect against some induced oxidative stress that causes various cancers and associated health problems. The administration of *O. sanctum* helps to regulated neurotransmitter levels which play an essential role in neuronal function [18]. On the *in vitro* studies, OSE was able to maintain the stability of ChAT expression at human cerebral microvascular endothelial cells (HCMECs) mimic young age and restore ChAT expression at mimic old age on *in vitro* model [21]. Another evidence stated that OSE exhibits neuroprotective effects against H_2_O_2_ induced neuronal cell damage in SH-SY5Y neuronal cells [22].

Human embryonic kidney-293 (HEK-293) is a cell line with the similar molecular pattern to widely used neuronal lineage [23]. HEK-293 expressed neurofilament, neuroreceptors, and neuron-specific metabolic enzyme [24]. However, there is still a limited study of the neuroprotective effect of OSE on HEK-293 cells. Trimethyltin (TMT) is a highly neurotoxic organotin compound that often used to induce neurodegeneration on the limbic system of *in vivo* and *in vitro* model [25]. In this present study, we develop *in vitro* experimental model of neurodegenerative diseases using HEK-293 cells and TMT as a neurotoxic agent to induce neurodegenerative diseases. This research aims to analyze the neuroprotective effect of OSE on HEK-293 cells as the *in vitro* model of neurodegenerative diseases.

## Materials and Methods

### Ethical approval

The use of all preclinical research material was approved by the ethics committee of Universitas Gadjah Mada, Yogyakarta, Indonesia, number: 00060/04/LPPT/IX/2016 (issued on September 26, 2016).

### Crude and OSE

*O. sanctum* leaves prepared from Center for Research and Development of Medicinal Plants and Traditional Medicines, Ministry of Health in Tawangmangu, Central Java, Indonesia. Crude extracts and OSE prepared as previously described [21].

### Maintenance of HEK-293 cell line

HEK-293 was a generous gift from Dr.rer.nat. Sentot Santoso (Giessen, Germany) and cultured in DMEM with FBS supplementation (DMEM; Lonza, Basel, Switzerland). Cells were divided into five groups, consist of healthy cells (normal, non-treated) as positive control (NT), cells treated with Camptothecin (CT) (Sigma, Steinheim, Germany) 500 μM act as negative control (CT), cells treated with TMT (Sigma, Steinheim, Germany) 10 μM (TMT), cells treated with OSE 75 μg/ml, and cells pre-treated with OSE 75 μg/ml then given TMT 10 μM (OSE+TMT). Then each of five group consists of three sample (n=3) we performed the experiments on 3 times different experiments as it appropriate for statistical analysis.

### MTT assay

We performed MTT assay to analyze the cell viability as previously described [26]. HEK-293 cells were trypsinized, 2.5×10^4^/well counted and seeded in each well of a 96-well plate. Cells were cultured in 200 μl DMEM to confluency. MTT assays were performed 24 h post application of OSE 75 μg/ml followed by 10 μM TMT. In some condition treatment with CT 500 μM was run as a negative control of viability meanwhile normal and OSE 75 μg/ml application act as positive control proliferation. MTT reagent (Sigma, Steinheim, Germany) was prepared to get the final concentration 5 mg/ml. Aspirate the medium, wash with PBS, and add MTT reagent (5 mg/ml) 100 μl for each well, including the blank medium. Incubate the cells for 2 h in a CO_2_ incubator. After incubation, formazan production was analyzed at 595 nm using fluorometer. Each sample was processed as 3-fold preparation. The percentage of viable cells was calculated relational to control cells using the following formula:

(Test sample absorbance – background absorbance)/(control absorbance – background absorbance) ×100%.

### Phase contrast microscopy

HEK-293 cells were trypsinized, and 2.5×10^4^ cells were seeded in each well of a 96-well plate. After 24 h cells were cultured in 200 μl DMEM in the presence of CT 500 μM, 10 μM TMT, 75 μg/ml OSE, and combination OSE and TMT. Then, HEK-293 cultured cells were observed using a phase contrast microscope (Nikon, Japan) and image was processed with a computerized image analysis system (Optilab, Yogyakarta, Indonesia).

### Reverse transcription polymerase chain reaction (RT-PCR)

Total RNAs from cultured cells were isolated using RNeasy Mini Kit (Qiagen, Hilden, Germany) according to manufacturer’s protocol. 100 ng/μl RNAs were used for reverse transcription PCR using Transcriptor One-Step RT-PCR (Roche) with specific primer. Primers used for ChAT were as follows: forward: 5’-CAACCATCTTCTGGCACTGA-3’, reverse 5’-TAGCAGGCTCAATAGCCATT-3’ [27]. PCR products were visualized in 2.5% agarose gel, for the quantification of DNA band density, data were imported as TIFF files into ImageJ (http://imagej.nih.gov/ij/). Glyceraldehyde-3–phosphate dehydrogenase (GAPDH) was also run as an internal control with primer compositions is as follow: forward: 5’-TGCACCACCAACTGCTTAGC-3’, Reverse: 5’-GGCATGGACTGTGGTCATGAG-3 [28].

### Statistical analysis

Multiple statistical comparisons were made using one-way ANOVA following by Tukey’s *post-hoc* test for MTT assay and ChAT expression data analysis. p<0.05 was assumed to represent statistical significance. The statistical analysis was performed using GraphPad Prism 6 (La Jolla, CA, USA).

## Results

### OSE increases the viability of HEK-293

**Figure-1:**
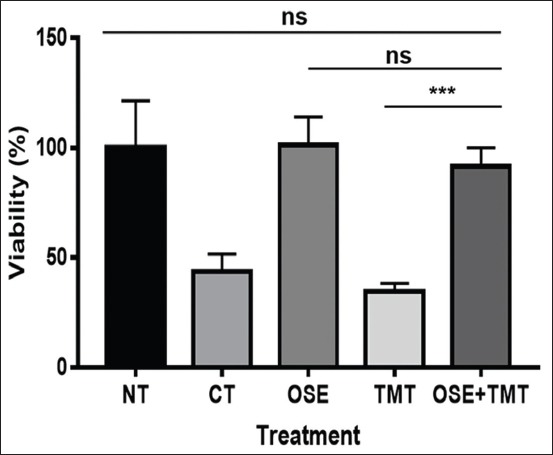
The OSE increased the viability of human embryonic kidney-293 (HEK-293) cells mimicking neurodegenerative diseases condition visualized by MTT analysis. After OSE, HEK-293 cells were induced by TMT and incubated for 24 h, MTT reagent (5 mg/ml) added 100 μl for each well, including the blank medium. Incubate the cells for 2 h in CO2 incubator. The formazan production was analyzed at 595 nm (NT: Non-treated, normal cells; CT: Camptothecin treated cells; OSE: *Ocimum sanctum* ethanolic extract-treated cells; TMT: Trimethyltin treated cells; and OSE+TMT: Cells given pre-treatment with *Ocimum sanctum* ethanolic extract-treated cells than induced by TMT). Statistical analysis was performed by one-way ANOVA followed by Tukey’s *post hoc* test as appropriate. Values are expressed as mean±standard deviation, n=3. *** p<0.05 shows significant differences with confidence interval 95%, n.s: non-significant.

MTT assay analysis on the HEK-293 shows that pre-treatment with OSE significantly increased the viability of TMT-induced apoptotic cells (p<0.05) (Figure-1). Normal cells (NT) were run as a positive control, and CT-treated cells acted as a negative control. From the graph (Figure-1), TMT has stronger toxicity to induce cell death than CT that results in the low percentage of cell viability on TMT groups. On the other hand, OSE administration could maintain the cell viability of OSE+TMT group even after induced by TMT. However, OSE treatment did not give significant effect to the viability of the normal HEK-293 cells on OSE group (p>0.05), it means the extract did not have the cytotoxic effect to the NT.

**Figure-2:**
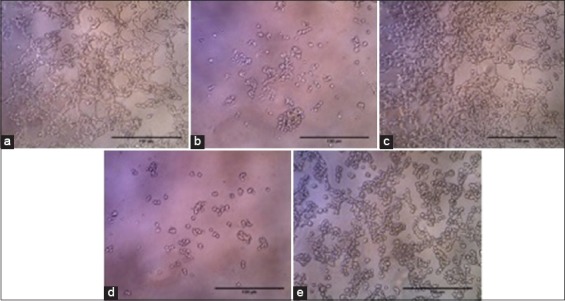
Photomicrograph of human embryonic kidney-293 (HEK-293) cells after 24 h incubation with and without the presence of *Ocimum sanctum* ethanolic extract (OSE) using a phase contrast microscope. (a) Normal HEK293 cells; (b) HEK-293 cells treated with Camptothecin (CT; (c) HEK-293 cells treated with OSE; and (d) HEK-293 cells treated with trimethyltin (TMT) (e) HEK-293 cells pre-treatment with OSE then challenged with TMT (10× objectives, scale bar 100 µm).

These results are also confirmed by the photomicrograph of HEK-293 cells using a phase contrast microscope, and it is proven that OSE administration maintains HEK-293 cells viability (Figure-2). The TMT (Figure-2d) and CT (Figure-2b) induced HEK-293 cells exhibited cell shrinkage and reduced the number of viable cells. However, OSE pre-treatment of HEK-293 cells on OSE+TMT group significantly protect the cells from TMT-induced cell damage (Figure 2e) and maintain the cells in the excellent condition as well as the non-treated (Figure 2a) and OSE treated cells (Figure 2c). There were no differences in cells morphology between normal HEK-293 groups.

### OSE stabilizes ChAT expression in TMT-induced HEK-293 cells

**Figure-3:**
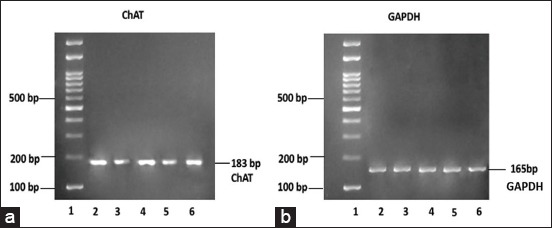
The expression of choline acetyltransferase (ChAT) on the human embryonic kidney-293 cells mimicking neurodegenerative conditions before and after treatment with *Ocimum sanctum* ethanolic extract (OSE). The RNA was isolated from cells lysate and amplified using reverse transcription polymerase chain reaction (RT-PCR). The ChAT RT-PCR product (183 bp) was visualized on 2.5% agarose gel (a) Glyceraldehyde-3–phosphate dehydrogenase was run as an internal control of the RT-PCR reaction (165 bp) (b). (1: Marker; 2: Non treated; 3: Camptothecin; 4: OSE; 5: TMT: Trimethyltin; and 6: TMT+OSE).

**Figure-4:**
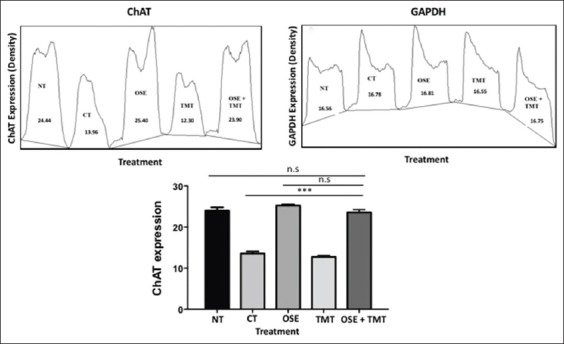
The ChAT density analysis. The results of DNA electrophoresis then transferred into TIFF and continued to analyze using Image J to define the density of DNA band which visualized the expression of ChAT. (a) Plot lanes of ChAT density (percent), (b) plot lanes of Glyceraldehyde-3–phosphate dehydrogenase density (percent), and (c) the graph of ChAT expression, statistical analysis was performed by one-way ANOVA followed by Tukey’s *post hoc* test as appropriate. Values are expressed as mean±standard deviation, n=3. ***p<0.05 shows significant differences with confidence interval 95%, n.s: non-significant.

The expression of functional genes associated with the cholinergic system, ChAT, which responsible for ACh synthesis is analyzed using RT-PCR. The agarose gel electrophoresis of RT-PCR results (Figure-3) showing sample number 3 (CT) and number 5 (TMT) has a thinner band than the other three groups. This indicates there is decreased ChAT expression on CT and TMT groups. Sample number 4 (OSE) shows a thicker band than sample number 1 (NT) and number 6 (OSE+TMT). This points out that there is an increased ChAT expression on HEK-293 cells with *O. sanctum* Linn. extract administration. This is further examined using ImageJ densitometry analysis to quantify the ChAT expression. The densitometry analysis of ChAT expression visualized plot lines and label peaks (Figure-4a and b). ChAT expression analysis showed higher peak and percent density on normal HEK-293 cells (NT), OSE treatment cells, and OSE+TMT treatment cells (Figure-4a). ImageJ gel analysis results described that TMT and CT groups show lower density than the other groups (Table-1). It means the ChAT expression markedly reduced on TMT and CT groups. Such alterations in the ChAT expression were restored with OSE administration before induced by TMT. We performed one-way ANOVA and Tukey’s *post-hoc* test to compare the means of percent density between groups (Figure-4c). The results show that administration of OSE on OSE group did not significantly increase ChAT gene expression and maintain the expression similar to NT group. ChAT expression on CT and TMT group significantly decreased in compared to the other groups. However, pre-treatment of OSE maintained the stability of ChAT expression on OSE+TMT group.

**Table-1:** Densitometry analysis of RT-PCR results using ImageJ, adjusted density is the final results of ChAT expression density after normalized by GAPDH.

Sample	ChAT		GAPDH		Adjusted density
	
	Percent	Relative density	Percent	Relative density	
NT	24.439	1	16.556	1	1
CT	13.963	0.6	16.783	1	0.6
OSE	25.400	1	16.806	1	1
TMT	12.295	0.5	16.546	1	0.5
OSE+TMT	23.902	1	16.754	1	1

NT=Normal cells, CT=Camptothecin, OSE=*Ocimum sanctum* ethanolic extract, TMT=Trimethyltin, RT-PCR=Reverse transcription polymerase chain reaction, ChAT=Choline acetyltransferase, GAPDH=Glyceraldehyde-3–phosphate dehydrogenase

## Discussion

Several herbal extracts have been shown to inhibit cell damage [29]. *O. sanctum* Linn. (Lamiaceae) is an Indonesian medicinal herb [21] and it is distributed worldwide [22]. It provides several function as an anti-inflammatory [12], antiallergic [13], antioxidant [14,30], radioprotective [31], and anticarcinogenic [32]. *O. sanctum* hydroalcoholic extract also showed a neuroprotective effect against H_2_O_2_ induced neuronal cell damage through its antioxidative defense mechanism [22].

HEK-293 is a cell line which derived in 1973 from primary human embryonic kidney cells transformed by sheared adenovirus 5 DNA [18]. It has a general molecular pattern of intermediate filament protein expression similar to that seen in PC12 cells and Ntera-2 cells, two widely used neuronal lineage cell lines [23]. Some findings already described that ChAT enzyme is not only expressed on neuronal cells but also on non-neuronal cells with barrier and immune functions [33,34]. HEK-293 also endogenously expressed muscarinic receptors [35] which are the marker of the cholinergic system. However, there is still controlled study about the neuroprotective effect of OSE in a cell line that mimics neuronal cell lineage.

Recently, also known as TMT is an organometal with potent neurotoxic effects by selectively damaging to the hippocampus, is used as a tool to create an experimental model of neurodegeneration [36]. TMT induced necrotic/apoptotic cell death [37] and oxidative stress [38]. In the present study, we have evaluated the protective effect of OSE against TMT challenge by MTT reduction assay in cultured HEK-293 cells. Some data proposed that excessive ROS formation regarded as a pathogenic factor during TMT induction caused the death of the cells [37]. On the other hand, OSE can maintain the cell viability by inhibiting lipid peroxidation, DNA damage, reactive oxygen species production, and depolarization of mitochondrial membrane [22]. Polyphenols and flavonoids are the main bioactive constituents of herbal extracts, and several studies have illustrated the neuroprotective and anti-stress of these materials [39].

Our current research shows on the MTT assay and phase contrast analysis, the presence of OSE may help the cell ability to still alive and proliferate (Figures-1 and 2). The intriguing question from this phenomenon is how the OSE could protect the cells? To answer the question, we run the ChAT gene expression analysis using RT-PCR. Surprisingly, we found that OSE pre-treatment can ameliorate ChAT expression on TMT-induced HEK-293 cells as well as the positive control (normal HEK-293 and OSE treatment only) (Figures-3 and 4, Table-1). It is already described that ChAT enzyme also plays a crucial role of neuroprotection in the cells. The depletion of the presynaptic cholinergic system is one of the causes of neurodegenerative symptoms, in which reduced activity of ChAT enzyme is observed [8,9]. The previous study indicates that administration of OSE promotes and restore the expression of ChAT on the deteriorated HCMECs. Therefore, it may give cells protection and help the production of ACh [21] and thus may promote significantly the viability of the cells. However, the treatment of OSE on normal HEK293 cells did not give significant impact to the appearance of ChAT. These results are in line with the low passage of HCMECs which did not significantly increase the expression of ChAT meanwhile in the high passage of HCMECs which mimics aging condition OSE promotes and restore the expression of ChAT [21]. This evidence also can explain that OSE maintains the stability of ChAT expression in normal HEK-293 cells (Figures-3 and 4; Table-1).

The role of ChAT is essential for ACh synthesis; the expression of ChAT has the linear effect to ACh production. Although ACh has been recognized for its role as a neurotransmitter and contributes in memory and cognitive process. It also has an autocrine function in various cell types. ACh has been exhibited to stimulate cytoskeleton organization, cellular proliferation, differentiation, and apoptosis [40-44]. Increasing of ACh stimulated the increasing of pyramidal cells density in CA1 and CA3 of the hippocampus in young- and middle-aged rats that also proved ACh as a regulator of neuronal cells neurogenesis [20]. In addition, the increased expression of ChAT also helps to improve and maintain the cognitive ability of the young- and middle-aged rats [26]. The blockade of nicotinic and muscarinic ACh receptors will lead to cellular dysfunction and cell death (apoptosis) [6]. The defect of a non-neuronal cholinergic system that disrupts the neurotransmission may involve in the pathogenesis of the diseases [21]. Therefore, ChAT and ACh are necessary to the neuroprotection mechanism of neuronal and nonneuronal cholinergic cells.

Based on our findings, we can prove that OSE can be a candidate of neuroprotective substance to increase the viability of HEK293 cells by maintaining ChAT expression to prevent neurodegenerative symptoms. It will be more intriguing to explore the role of ChAT in another cellular signaling process to understand the whole of neuroprotection mechanism comprehensively. However, this research is based on *in vitro*; it needs the determination of proper dosage of this extract to be performed on *in vivo* model that can resemble the physiological mechanism on a human.

## Conclusion

This result indicates that OSE has a neuroprotective effect against TMT by increasing cells viability and maintaining ChAT expression of HEK-293 as *in vitro* model of neurodegenerative diseases.

## Authors’ Contributions

DLK and HW designed the experiments and study; PH and MBAM performed the experiments; PH, NW, DLK, and HW interpreted the data; PH, DLK, and HW wrote the manuscript. All authors read and approved the final manuscript.
